# Plant Diversity and Sustainable Landscape Management: The Case of Misiliscemi, a New Municipality in Sicily

**DOI:** 10.3390/plants14040548

**Published:** 2025-02-10

**Authors:** Michele Aleo, Giuseppe Bazan

**Affiliations:** 1 Independent Researcher, Via S. Safina 1, 91100 Misiliscemi, Italy; michele.aleo@libero.it; 2 Department of Biological, Chemical and Pharmaceutical Sciences and Technologies (STEBICEF), University of Palermo, Via Archirafi 38, 90123 Palermo, Italy

**Keywords:** vascular flora, floristic knowledge, floristic studies, primary biodiversity data, habitat, landscape, biodiversity conservation, environmental planning, mediterranean

## Abstract

Floristic and biodiversity knowledge play a crucial role in ecosystem conservation and sustainable land management, particularly in urban-rural contexts that can serve as biodiversity reservoirs, hosting species of high biogeographic value. Focusing on the new municipality of Misiliscemi, established in 2021 in Sicily and now facing the challenge of developing new management strategies, this study provides fundamental knowledge on the plant biodiversity of the area and explores how the integration of floristic and environmental data can guide territorial planning strategies aimed at preserving natural capital and ecosystem services. The research, based on field surveys conducted over many years, taxonomic identification of species, analysis of biological forms and chorological data, evaluation of ecological indicators, and GIS-based habitat mapping according to the EUNIS classification, has made it possible to obtain a comprehensive dataset. The results of this work led to the identification of 623 taxa, recording new findings for the Sicilian flora, including both native and alien species, which represent primary biodiversity data crucial for plant resource management. In addition, 42 habitat types were mapped, highlighting that approximately 80% of the territory is occupied by vegetated man-made habitats. Despite anthropogenic pressures and landscape modifications, Misiliscemi retains significant plant biodiversity, including habitats and species of conservation interest, that represent a vital resource for natural capital and ecosystem services. This knowledge base, in addition to constituting the scientific foundation upon which this young municipality can develop an urban planning strategy aimed at achieving sustainable local development, also represents a methodological approach that highlights how basic knowledge of urban biodiversity should be considered a crucial aspect of sustainable urban planning worldwide.

## 1. Introduction

Plants are essential components of ecosystem functioning; they are key elements in the definition of habitats, and for this reason, their study is the first step towards the interpretation of the biological and environmental characteristics of a territory. One of the most common methods for studying plant diversity involves field surveys, in which information on the flora and vegetation of a territory is collected, leading to the drafting of floristic and vegetation inventories that serve as primary biodiversity data. These data, in addition to being essential for understanding the ecological framework and the landscape of a given area, are recognized as a tool for developing conservation strategies and measures, as well as for the sustainable management of natural heritage at both local and global levels [1,2,3]. Furthermore, understanding plant diversity through field studies provides a foundation for integrating biodiversity conservation into urban and landscape planning, particularly in urban-rural systems. Urban and landscape planning plays a key role in translating knowledge on biodiversity into strategies and measures to achieve sustainability goals within anthropogenic landscapes, including urban-rural systems (cities, towns, suburbs, and rural areas), which often serve as valuable reservoirs of biodiversity, hosting species of high biogeographic value that are not found elsewhere [4,5].

This new approach to planning has led botanists, over the past decades, to increasingly focus on rural and urban systems and the role they can play in biodiversity conservation, both at the European and Italian levels (see Domina et al., 2020 [6] for a review).

Preserving biodiversity and urban-rural systems is of paramount importance, considering that artificial and agricultural systems occupy 3.37% and 33.36% of Europe’s surface, respectively [7], and that 38.9% of the EU population lives in cities, 35.9% in towns and suburbs, and 25.2% in rural areas [8]. In urban-rural contexts, the conservation of plant species, ecosystems, and their associated landscapes is crucial for sustaining natural capital and the ecosystem services essential to human well-being [9].

Floristic diversity knowledge, therefore, must be incorporated into urban and regional planning to ensure the maintenance of ecosystem services provided by biodiversity [10].

Starting from these premises, floristic diversity and habitats of the Misiliscemi area, located in the province of Trapani (western Sicily), were examined. Misiliscemi is the most recently established municipality in Italy, having been officially recognized in 2021. The newly formed local administration, tasked with developing a territorial planning strategy from scratch, must take into account the natural and environmental values of the area and acquire data on the local flora and habitats. The flora of this part of Sicily has been the subject of floristic studies since the early 20th century. Among the most significant contributions are those of A. Ponzo, who, building on data reported by Gussone [11,12] and Lojacono [13,14,15,16,17] for the entire region, provided a unified overview of the flora of the Trapani area through numerous works published during the first half of the 20th century [18,19,20,21,22,23]. In the following years, only a few studies have expanded knowledge of this area [24,25,26,27]. However, a detailed and specific knowledge framework for the Misiliscemi area is still lacking, which could be valuable for planning purposes.

The aim of this study is to describe and assess the plant diversity of the territory of Misiliscemi, explore the potential role of plant diversity as a key indicator of natural capital of the territory, and provide insights for urban planning strategies that preserve and enhance the floristic diversity. This body of knowledge not only serves as the scientific foundation for this newly established municipality but also exemplifies a methodological approach that underscores the importance of understanding urban biodiversity as a key component of sustainable urban planning on a global scale.

## 2. Materials and Methods

### 2.1. Study Area

**Figure 1:**
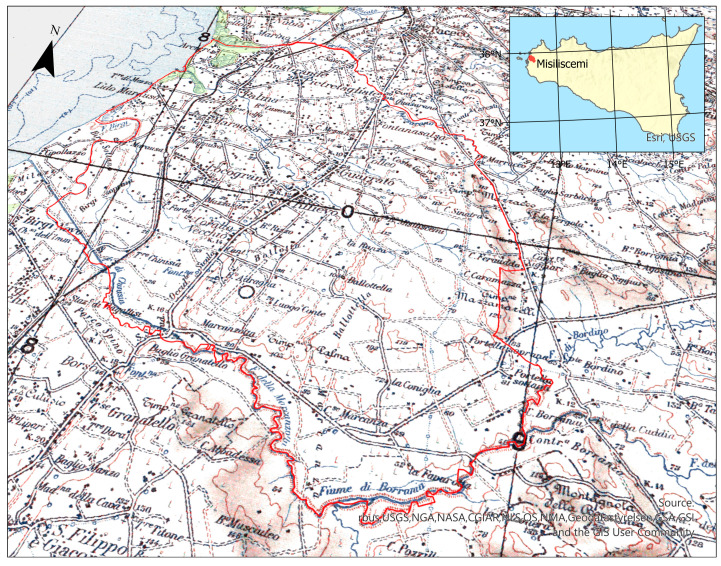
Location of the study area and topographic map from the Italian Geographic Military Institute [30], with the boundaries of the municipality of Misiliscemi outlined in red and the Natura 2000 Network highlighted in green. The place names mentioned in the text correspond to those shown on this map.

Misiliscemi is a municipality in the province of Trapani (western Sicily), officially established in 2021 by detaching eight urban nuclei (hamlets) previously under the jurisdiction of Trapani: Rilievo, Fontanasalsa, Guarrato, Locogrande, Marausa, Palma, Salinagrande, and Pietretagliate (Figure 1). 

The municipality covers an area of 9253.71 [28] hectares, stretching from low hills (127 m a.s.l.) to the coast, forming an urban system of small settlements dispersed within an agricultural matrix [29].

The name “Misiliscemi” originates from the toponym referring to the “Baglio Misiliscemi,” located approximately 7 km from the coast near the “Misiliscemi” stream, which traverses the municipality from east to west. The term Misiliscemi derives from the Arabic Manzil as-Sayl (منزل السيل), meaning “house of the stream,” referencing the medieval Islamic settlement once located at the site of the current baglio.

The ecological and environmental setting of the area is defined by a Mediterranean climate, characterized by mild winters and hot, dry summers. Data from the nearby Trapani weather station show an average maximum temperature of 21.7 °C, an average minimum of 14.4 °C, and a mean daily temperature of 18.1 °C, with an average daily range of 7.3 °C [31]. Annual rainfall is approximately 483 mm, distributed over 64 rainy days. The wettest months are October (71.1 mm), November (69.6 mm), and December (75.1 mm), whereas June (8.0 mm), July (1.7 mm), and August (9.5 mm) are the driest months. According to Rivas-Martínez’s [32] bioclimatic classification, the study area falls within the Thermo-Mediterranean bioclimate with a lower sub-humid ombroclimate [33].

From a lithological perspective, the municipality exhibits significant geodiversity, as it is characterized by five lithostratigraphic units that contribute to a variety of soils and, consequently, to different ecological and agricultural potentials. The lithostratigraphic units are: (1) Recent deposits; (2) clastic carbonates (Pleistocene–Late Pliocene); (3) reef carbonates (Early Messinian) and the Terravecchia Formation (Early Messinian–Late Tortonian); (4) marly arenaceous members (Langhian–Early Tortonian); (5) biocalcarenites and arenaceous strata (Mischio, Early Miocene) [34]. These calcareous and arenaceous terrains define a gently undulating lowland platform, which inclines slightly inland to an elevation of about 120 m, interrupted by wide, shallow valleys carved by streams such as the Misiliscemi Torrent and the Birgi River. These plains gradually transition into the coastline, often bordered by narrow beaches flanked by terraces or small coves, where historical salt pans (e.g., Salina Grande, Salina San Francesco, Salina Fiume) were established. The predominant soil types on the recent alluvial deposits are alluvial soils, which exhibit vertic characteristics in predominantly clayey textures. On the calcarenite platform, red soils are the most common [35].

The productive capacity varies depending on different pedological conditions, primarily in relation to soil texture. However, it can generally be considered good, as evidenced by the agricultural use of the land.

**Figure 2:**
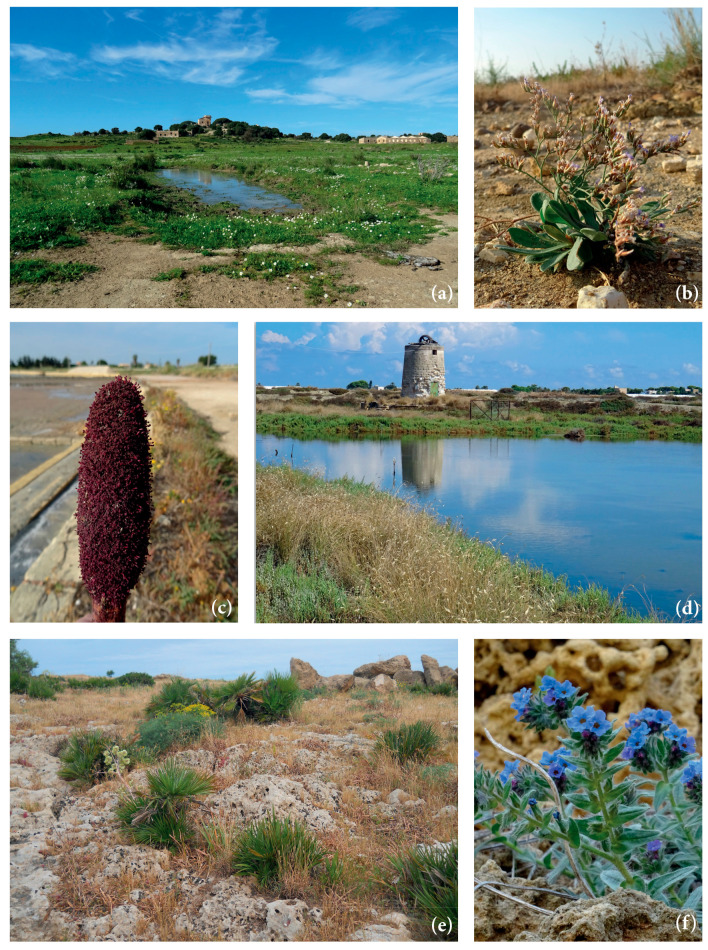
(**a**) Ephemeral (brackish) wetland environments in the area of former Chinisia military airport; (**b**) *Limonium dubium* in the area of the Chinisia area; (**c**) *Cynomorium coccineum* at the edges of the basins of the Salina San Francesco; (**d**) Halophilous vegetation aspects in the Salina San Francesco (foreground: *Lygeum spartum*); (**e**) Garrigue with *Chamaerops humilis* in the Rocche Draele; (**f**) *Alkanna tinctoria* on calcarenitic substrate.

The landscape is heavily influenced by agricultural activity, with widespread olive groves, vineyards, scattered orchards, arable fields, and irrigated gardens, creating a complex mosaic interspersed with numerous rural houses and small urban nuclei connected by a dense network of generally straight roads [36]. Pastures and uncultivated areas provide habitat for local biodiversity, with many small fragments of natural vegetation scattered throughout the territory. Notably, the abandoned Chinisia military airport, still designated as a military area, has preserved a large undeveloped surface. This area has remained free from urbanization and agricultural activities, serving as a refuge for habitats and diverse flora and fauna species. Particularly significant are the rare inland saline habitats (Figure 2a), which host uncommon species such as *Limonium dubium* (Refer to Table S1 for the authors of the taxa). (Figure 2b).

The municipal territory of Misiliscemi includes the Special Area of Conservation (SAC) ITA010012 “Marausa, macchia a *Quercus calliprinos*”, a site covering a small area of 0.719 hectares, which preserves a significant relict fragment of forest vegetation dominated by spiny oak (*Quercus coccifera* L.), phytosociologically classified as *Chamaeropo-Quercetum calliprini* [25]. The site hosts the priority habitat 6220* as well as habitat 5330 [37]. Furthermore, part of the coastline within the study area lies within the SAC ITA010007 “Saline di Trapani” (Figure 2c,d) which encompasses coastal, dune, and halophilous vegetation corresponding to habitats 1150*, 1210, 1420, 1510*, and 6220*.

The coastal area of the salt pans is of significant naturalistic importance for birdlife associated with salt production activities. Although the salt pans are artificial environments modified and managed by humans, they provide particularly favorable conditions for birds. The basins, characterized by varying salinity and depth, offer diverse ecological niches that cater to the needs of both resident and migratory species. For this reason, the site is designated as an Important Bird Area (IBA) and a Special Protection Area (SPA) under the Birds Directive (2009/147/EC).

### 2.2. Field Surveys and Data Analysis

The compilation of the floristic inventory was carried out through a repeated series of field surveys spanning over two decades (2000–2024). The spontaneous vascular flora was recorded, including both naturalized and occasionally alien species. The explorations began with the aim of searching for botanical curiosities and new species, initially following a non-systematic approach. However, over time, this led to a comprehensive exploration of the entire territory across different seasons. While this method has the limitation of not allowing a spatial comparison of floristic surveys, it still provides valuable information on species presence and absence.

Species identification was primarily based on Pignatti [38] and Pignatti et al. [39]. In critical cases, comparisons were made with exsiccata preserved in the Botanical Garden Herbarium of Palermo (PAL). Additionally, the publication of photographs of many taxa on the Acta Plantarum forum (www.actaplantarum.org) proved useful, as it enabled comparisons with other scholars and botany enthusiasts in cases of doubt.

The nomenclature of taxa follows Bartolucci et al. [40] and Galasso et al. [41], as well as the Portal to the Flora of Italy [42].

Taxa that became extinct in the territory during these decades of surveys due to land transformations and anthropogenic pressures (primarily fire) were marked with the symbol “†”

For the taxonomic analysis and standardization of the data related to the studied flora, an in-depth search of taxonomic information was conducted using the taxize package in R [43]. This package allows access to taxonomic and phylogenetic data from authoritative online databases such as Plants of the World Online (POWO), GBIF, and ITIS.

To analyze phylogenetic relationships at the species level for native taxa, the R package V.PhyloMaker2 [44] was used. Among the species in the dataset, a phylogenetic tree was constructed based on the megaphylogeny GBOTB.extended.TPL, which relies on The Plant List for standardized nomenclature. The species in the dataset were integrated into the reference tree using Scenario S1, which exclusively considers species already present in the megaphylogeny without adding hypothetical clades for missing species. This approach ensures phylogenetic inference based on robust data, minimizing the introduction of taxonomic uncertainties. The generated tree was visualized in a circular layout using the ggtree package in R [45].

This methodology enabled the exploration of phylogenetic relationships among the studied species and provided a visual representation consistent with the available taxonomic information.

Biological forms and chorotypes were attributed according to Pignatti et al. [39]. The chorotypes were simplified as follows: Alien, Endemic, Eurasian, Euro-Mediterranean, Mediterranean, Steno-Mediterranean, and Wide Distribution. For each species, the habitat where it was observed or collected was reported.

The Ecological Indicator Values (EIVs) of Ellenberg [46], as adapted to the Italian flora by Pignatti et al. [47] and updated when available [48], were used to describe the ecological requirements of the taxa included in the checklist and to provide an overall assessment of the entire flora. EIVs were calculated at the species level, meaning that different subspecies of the same species share the same EIVs.

The habitat map was created using the Nature Map [49] as the base data, reclassified according to the The EUNIS (European Nature Information System) Habitat Classification [50]. All cartographic elaborations were carried out in a GIS environment using the ArcGIS Pro package [51].

## 3. Results

### 3.1. Floristic Surveys and Data Analysis

**Figure 3:**
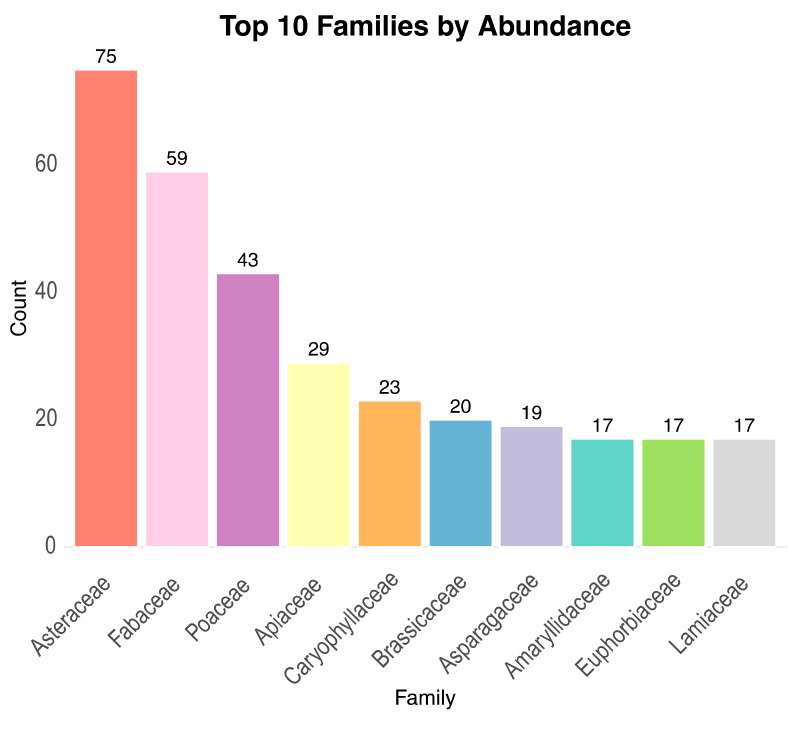
The most representative families of the flora of Misiliscemi.

**Figure 4:**
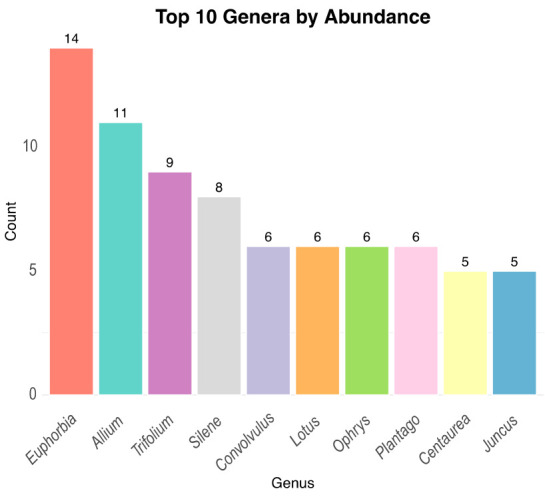
The most representative genera of the flora of Misiliscemi.

The floristic inventory of Misiliscemi includes 623 taxa (Table S1), from which 471 were reported at the species level, 160 at the subspecies level and 1 at the varieties level. Taxa are distributed across 38 orders, 93 families, 378 genera. From families, those with the highest number of taxa were Asteraceae (75), Fabaceae (59), Poaceae (43), Apiaceae (29), Caryophyllaceae (23), Brassicaceae (20), Asparagaceae (19) (Figure 3). The genera with more taxa are *Euphorbia* (14), *Allium* (11), *Trifolium* (9), *Silene* (8), *Convolvulus* (6), *Lotus* (6), *Ophrys* (6), *Plantago* (6), *Centaurea* (5) and *Juncus* (5) (Figure 4).

**Figure 5:**
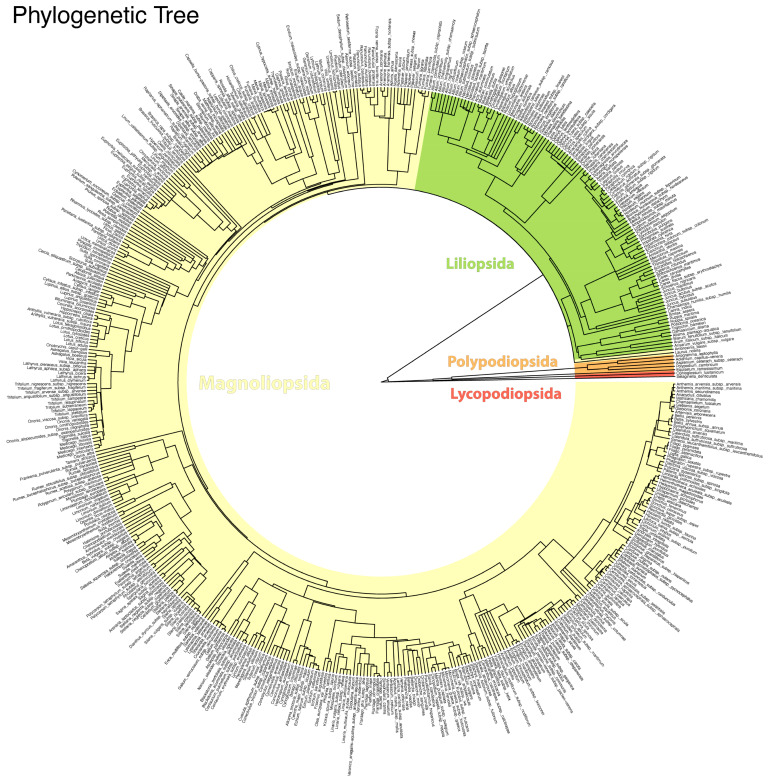
Phylogenetic tree of the 537 native species included in the checklist of Misiliscemi. Classes, specific, and infraspecific taxa are labeled.

The entire flora was dominated by Magnoliopsida (77.0% of the taxa), followed by Liliopsida (21.3%), Polypodiopsida (1.0%), Pinopsida (0.5%) and Lycopodiopsida (0.2%) (Figure 5).

The floristic study resulted in a new record for the Sicilian flora: *Blackstonia acuminata* subsp. *acuminata*. This species was collected at the Chinisia site (37.889000° N, 12.535432° E) in a Mediterranean dry grassland.

**Figure 6:**
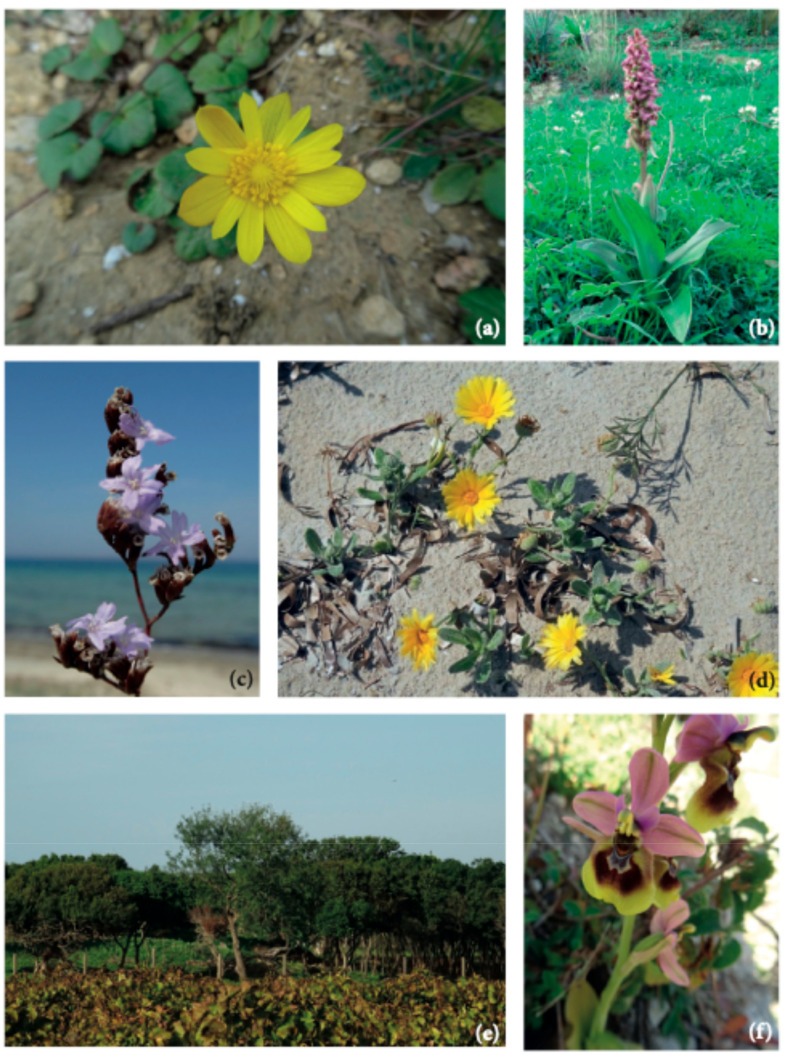
(**a**) *Anemone palmata* in the Marcazzotta area; (**b**) *Barlia robertiana* observed within a formation of dwarf palm (*Chamaerops humilis*); (**c**) *Limonium virgatum* in the brackish zones of Salina Grande; (**d**) *Calendula suffruticosa* subsp. *maritima* on the Marausa beach; (**e**) Relict aspects of the *Quercus coccifera* formation (*Chamaeropo-Quercetum calliprini*) in Marausa; (**f**) *Ophrys tenthredinifera* in the garrigues of the former Chinisia airport.

Among the species of conservation concern recorded in the study area are *Anemone palmata* (Figure 6a), *Anthemis secundiramea*, *Anacamptis collina, Barlia robertiana* (Figure 6b), *Limonium virgatum* (Figure 6c), *Calendula suffruticosa* subsp. *maritima* (Figure 6d), *Cressa cretica*, *Cynomorium coccineum* subsp. *coccineum* (Figure 4), *Daucus carota* subsp. *drepanensis*, *Eryngium tricuspidatum* subsp. *bocconei*, *Galium verrucosum* subsp. *verrucosum*, *Ophioglossum lusitanicum*, *Limonium densiflorum*, *Limoniastrum monopetalum*, *Quercus coccifera* (Figure 6e), *Ophrys tenthredinifera* (Figure 6f), *Orchis italica*, *Serapias cordigera* subsp. *cordigera, S. lingua, S. parviflora, S. vomeracea, Triglochin barrelieri*.

Five species have gone extinct in the territory over the past twenty years: *Cistus creticus* subsp. *creticus*, *C. salviifolius*, *Cytinus hypocistis* subsp. *hypocistis*, *Erica multiflora* subsp. *multiflora*, and *Thymelaea hirsuta*.

Among the significant species recorded, special mention must be made of *Quercus coccifera* (Figure 6e), the only Fagaceae species found in the area. It represents a relict element of the natural forest vegetation in this region. Among the most representative species of Mediterranean maquis are several sclerophylls, including *Pistacia lentiscus*, *Chamaerops humilis* (Figure 2e), *Rhamnus lycioides* subsp. *oleoides, Phillyrea latifolia*, *Teucrium fruticans*, and *Olea europaea* var. *sylvestris*. These species have been preserved on the calcarenite substrates in the Rocche Draele area and on the low hills of the Ballottella district, where the three historic “bagli” (farmhouse complex) of Misiliscemi, La Runza, and Ballottella are located (Figure 1).

The rocky habitats also host several interesting chasmophytes, such as *Capparis spinosa* subsp. *spinosa*, *Dianthus illyricus* subsp. *haynaldianus*, *Ruta chalepensis*, *Centranthus calcitrapae* subsp. *calcitrapae, Petrosedum sediforme* subsp. *sediforme,* and *Umbilicus horizontalis.* Additionally, these habitats provide refuge for species such as *Alkanna tinctoria* (Figure 2f), *Cytisus infestus* subsp. *infestus*, *C. laniger*, *Daphne gnidium*, *Globularia alypum*, *Micromeria graeca* subsp. *fruticulose*, *M. graeca* subsp. *graeca*, *M. nervosa*, *R. alba* subsp. *alba*, *Stachys major*, and *Thymbra capitata*.

### 3.2. Life-Forms and Chorological Spectrum

**Figure 7:**
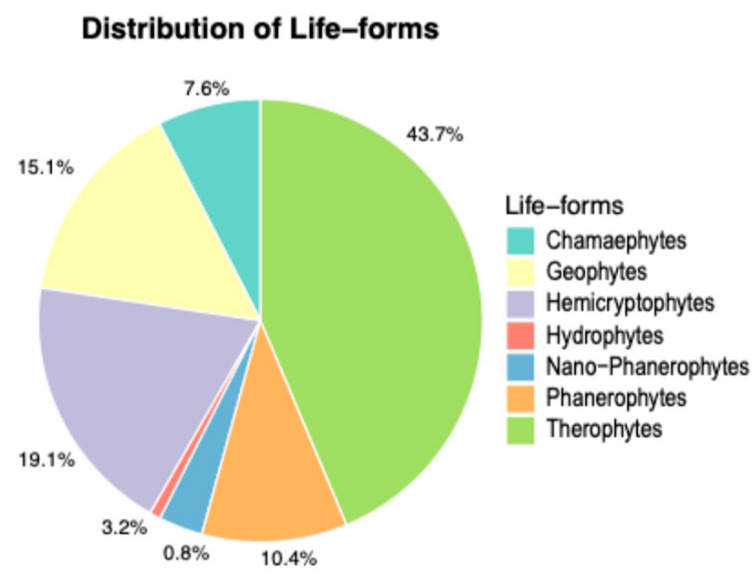
Biological spectrum of the flora of Misiliscemi.

The analysis of the biological spectrum shows that the most represented species are Therophytes (43.7%), Hemicryptophytes (19.1%), Geophytes (15.1%), Phanerophytes (10.5%), Chamaephytes (7.6%), Nano-Phanerophytes (3.2%), and Hydrophytes (0.8%) (Figure 7). The most abundant subforms are scapose therophytes (T scap: 243), scapose hemicryptophytes (H scap: 60), bulbous geophytes (G bulb: 57), rhizomatous geophytes (G rhiz: 36), suffruticose chamaephytes (Ch suffr: 34), and shrubby phanerophytes (P caesp: 27). The H/T ratio (a bioclimatic index) is 0.44.

**Figure 8:**
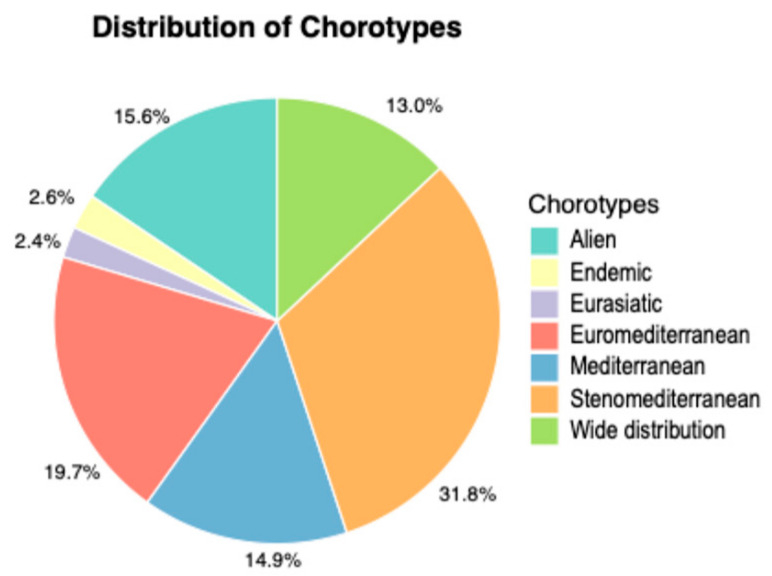
Chorological spectrum of the flora of Misiliscemi.

Regarding chorological types, Stenomediterranean taxa dominate with 199 species (31.9%), followed by Euromediterranean taxa with 122 species (19.6%), Mediterranean taxa with 93 species (15.0%), Wide distribution taxa with 81 species (13.0%) and Eurasiatic taxa with 14 species (2.3%) (Figure 8). Alien species account for 97 taxa (15.6%), of which 41 are considered invasive and 56 are naturalized (17 casual). Among the alien species, 25 are archaeophytes, while 72 are neophytes.

Two taxa are new records for Italy: *Dovyalis caffra* and *Aloe × delaetii*. *Euphorbia serpens* subsp. *fissistipula* and *Hibiscus rosa-sinensis* is reported for the first time in Sicily. Among invasive alien species of Union concern, listed under Regulation [EU] 1143/2014 and its updates, occurring in Misiliscemi are *Acacia saligna*, *Ailanthus altissima*, and *Cenchrus setaceus*. The endemic species total 16, of which 10 are Italian endemics, 2 are subendemics (Carlina sicula subsp. sicula and Crocus longiflorus), and 4 are Sicilian endemics: *Allium obtusiflorum, Echium italicum* subsp. *siculum*, *Romulea linaresii* subsp. *linaresii*, and *Silene crassiuscula*.

### 3.3. Ecological Indicators

**Figure 9:**
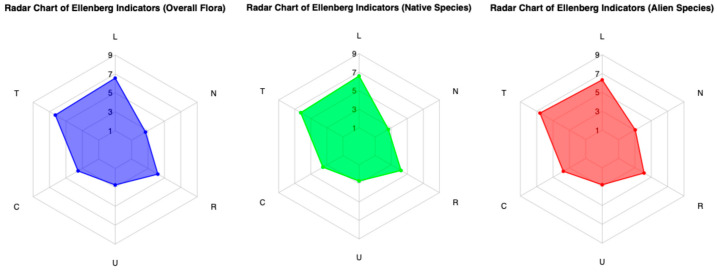
Radar charts of Ellenberg indicators for overall, native, and alien flora.

The Ellenberg Indicator Values (EIVs) of 535 species were considered, excluding all species for which values were unavailable (mostly cultivated species accidentally naturalized and/or alien). The mean values calculated for the entire dataset of indicators were 8.64 for Light (L), 8.28 for Temperature (T), 4.47 for Continentality (C), 3.4 for Moisture (U), 5.4 for Soil Reaction (R), and 3.33 for Nutrients (N) (Figure 9). A total of 95 alien species were evaluated. Data analysis highlights ecological differences between the overall analyzed flora, native species, and alien species (Table 1).

Native species (8.68) show higher light (L) values compared to the overall average (8.64), while alien species (8.33) exhibit lower values. Regarding Temperature (T), alien species (8.71) display higher values than both the overall average (8.28) and native species (8.22). For Continentality (C), native species (4.43) show slightly lower values compared to the average (4.47), whereas alien species (4.78) display significantly higher values.

In terms of Soil Moisture (U), native species (3.39) register slightly lower values compared to the average (3.40), while alien species (3.48) show higher values. Looking at Soil Reaction/pH (R), native species (5.42) have a higher average compared to the overall average (5.40), whereas alien species (5.29) fall below it. Finally, for Nutrient Availability (N), native species (3.26) show lower values, associated with nutrient-poor soils, compared to the average (3.33), while alien species (3.81) stand out with higher values, indicating a preference for more nutrient-rich soils.

### 3.4. Habitat

**Figure 10:**
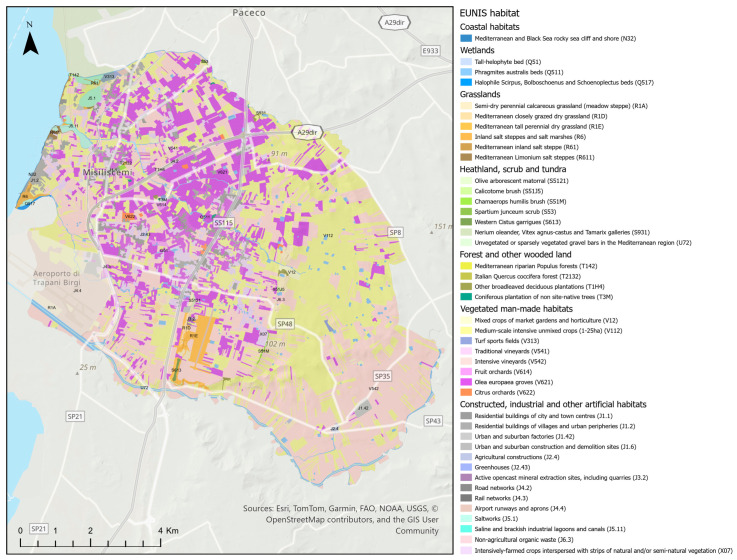
Map of the landscape types according to the EUNIS Habitat Classification.

The results obtained from GIS cartographic analyses and field validation allowed the identification of 42 EUNIS habitat types (Figure 10), divided into the main categories defined by the European classification: Coastal habitats (5.68 ha), Wetlands (222.38 ha), Grasslands (387.13 ha), Heathland, scrub and tundra (including Unvegetated or sparsely vegetated habitat) (38.61 ha), Forest and other wooded land (35.66 ha), Vegetated man-made habitats (7404.20 ha), and Constructed, industrial and other artificial habitats (1146.94 ha). The plant landscape is varied, predominantly characterized by anthropogenic types; in fact, most of the territory is covered by intensive vineyards (32.33%), intensive unmixed crops (29.65%), and *Olea europaea* groves (16.86%).

The most represented semi-natural environments are semi-dry perennial calcareous grasslands, which account for 2.51% of the study area. A detailed distribution of the surface areas for individual categories is provided in Table 2.

Within these vegetation types, 12 distinct habitats of interest have been identified (Directive 92/43/EEC), some of which are located outside the Natura 2000 Network (Table 3). Notably, the priority habitat 6220* is situated between the districts of Chinisia and Marcanzotta. This habitat hosts important species such as *Anacamptis collina*, *Anemone palmata*, *Anacamptis collina, Barlia robertiana*, *Blackstonia acuminata* subsp. *acuminata*, *Daucus carota* subsp. *drepanensis*, *Eryngium tricuspidatum* subsp. *bocconei*, *Globularia alypum*, *Orchis italica*, *Serapias cordigera* subsp. *cordigera*, *S. lingua*, *S. parviflora*, and *S. vomeracea*.

Equally significant are the ephemeral (brackish) wetland environments (Figure 2a), which flood during the winter season and support a remarkable hydro-halophilous flora, including *Carex caryophyllea, Damasonium alisma*, *Juncus hybridus*, *Limonium dubium, Lythrum junceum*, *Plantago crassifolia*, *Spergularia bocconei*, *Romulea ramiflora* subsp. *ramiflora*, *Romulea linaresii* subsp. *linaresii*, *Trifolium lappaceum* and *Ranunculus muricatus*.

## 4. Discussion

The municipality of Misiliscemi represents a paradigmatic element of the landscape of the western coastal plain of Sicily, characterized by an anthropized environment shaped for centuries by agriculture. This has created a mosaic of cultivated areas, predominantly composed of woody plantations, developing on fertile red soils. These are interspersed with calcarenite outcrops, locally known as “sciare,” which are characterized by low Mediterranean scrub with dwarf palm (*Chamaerops humilis*) or Mediterranean dry grasslands. The natural vegetation, consisting of formations referable to *Chamaeropo humilis-Quercetum calliprini* (on red soils) [52] and *Chamaeropo humilis-Oleetum sylvestris acanthetosum mollis* (on lithosols) [53,54,55], survives only in a few relict fragments. Despite centuries of natural resource exploitation, the land reclamation efforts between the late 19th century and the first half of the 20th century, which destroyed the marshy areas and wetlands in the area [56], it remains uncertain whether this landscape still retains part of its natural capital and the associated ecosystem services. How does the flora contribute to understanding the biodiversity component of natural capital in the Misilicemi territory?

The municipality of Misiliscemi has area with a limited surface (92.5 km²), accounting for 0.45% of Sicily’s total area, despite this, its flora represents 18.9% of the regional flora. The species/area ratio (S/A) of the Misiliscemi flora is 6.7, a metric commonly used to evaluate and compare floristic richness across different regions [57]. Comparing Misiliscemi to other case studies, such as the city of Palermo (63.5 km²), which includes the city center and suburban ex-agricultural areas, including the Favorita Park in the Mount Pellegrino Natural Reserve, the floristic diversity is 1052 taxa (S/A = 16.6) [6]. For the urban area of Naples (117.3 km²), De Natale and La Valva recorded 984 taxa (S/A = 8.39) [58]. More recently, in Empoli municipality (63.5 km²), Peruzzi cataloged 757 specific and subspecific taxa (S/A = 11.69) [59]. The comparison reveals that the floristic diversity in Misiliscemi is lower than that observed in other study areas, indicating the high anthropization of this territory. Most interesting taxa have been observed or collected along the edges of fields, in grazing areas, fallow lands, roadsides, and ruins. There are few semi-natural areas, represented by degraded secondary shrub formations.

The biological spectrum provides a clear picture of the structural composition of the vegetation cover, which consists of 77.9% therophytes, hemicryptophytes, and geophytes (Figure 6), species prone to frequent fires during the hot Mediterranean summers. Among the 66 phanerophytes, 39 taxa (approximately 60%) are alien species (cultivated and naturalized species). The low incidence of woody species is also confirmed by the cartographic analysis of habitats, which shows low coverage values for forest and shrub formations. (Figure 10, Table 2). When comparing the biological spectrum of the study area with that of the coastal area of the Province of Trapani recorded in previous studies [26,27], the prevalence of therophytes, hemicryptophytes, and geophytes (85% for the southern coast of the province and 70% for the northern coast of the province) confirms that Misiliscemi’s landscape reflects the landscape structure of the western Sicilian coast. The high incidence of therophytes is an indicator of significant disturbance, which leads to an increase in annual species or short-lived perennials [60]. In fact, comparing the T/H ratio of the vascular flora of Misiliscemi with that of the Monte Cofano Nature Reserve [61] located a few kilometers further north along the coast, reveals respective values of T/H = 2.3 (Misiliscemi) and T/H = 0.9 (Monte Cofano). This means that, in similar climatic contexts, the T/H > 1 value (indicative of a more altered environment) confirms that the high incidence of therophytes in Misiliscemi is correlated with anthropogenic disturbance and not with summer aridity, which is consistent across both areas. The chorological analysis of the flora highlighted the abundance of steno-Mediterranean and Mediterranean species. This result aligns with the bioclimatic and phytogeographical context of the area and is consistent with what is outlined by the biological spectrum. The chorological spectrum is comparable to that of other nearby areas investigated from a floristic perspective in western Sicily [27,62].

The high incidence of widely distributed and alien species (13% and 15.6%, respectively) serves as an indicator of anthropogenic disturbance in the area. These species are often favored by anthropogenic factors such as habitat modification, pollution, and intensive land management [63]. In particular, the alien species observed in the Misiliscemi area are mostly archaeophytes that act as crop weeds or species introduced as ornamentals, which subsequently spread in disturbed urban and rural environments. An example is *Aloe × delaetii*, likely a new alien entity for Italy [41], found in a small population in good vegetative condition in the saline coastal area of Marausa, probably having escaped cultivation. This horticultural hybrid was found alongside *Austrocylindropuntia subulata*, another species commonly cultivated in gardens. It is therefore assumed that their presence in the natural environment is due to the illegal garden waste dumping in semi-natural areas, triggering processes of biological invasion [64]. The same applies to *Hibiscus rosa-sinensis*, a small tree commonly cultivated in the gardens of Siciliy, which was incidentally found naturalized along the roadside. The risk of the spread of plants cultivated for ornamental and horticultural purposes is well known within the scientific community [65]; however, awareness of these issues is still lacking among the general population. Among the alien species, two uncommon taxa deserve special mention: *Dovyalis caffra* e *Euphorbia serpens* subsp. *fissistipula*, found in the locality of Marcanzotta.

The population of *D. caffra*, a small-size tree, native to South Africa, known to the authors for over two decades, remains stable at the edge of an olive grove and shows no signs of expansion. In 2023, *E. serpens* subsp. *fissistipula* was discovered in the territory of Misiliscemi. This South American species was introduced to Europe for commercial purposes and is reported as an adventive species in southern France and northwestern Italy (Liguria) [66]. Its presence represents a new record for the alien flora of Sicily, where only *Euphorbia serpens* Kunth was previously known [41]. These species, along with all other alien species, as is well known, cause various ecological and environmental problems, with direct impacts on native plant communities. They compete with native species for light, water, and nutrients, ultimately reducing local biodiversity [67]. Species such as *Ailanthus altissima*, *Cenchrus setaceus*, and *Carpobrotus acinaciformis* are highly invasive in the study area and require active control, especially within Natura 2000 sites. In many cases, for alien species introduced long ago, we are no longer able to assess the impact they have had on native species. One of the most emblematic cases in the territory is represented by *Oxalis pes-caprae*, introduced at the end of the 18th century. It is now invading ruderal areas, agricultural lands, grasslands, and old-field habitats, coloring the winter landscape yellow. Another interesting case is that of *Arundo donax*; this archaeophyte is one of the most invasive plant taxa in subtropical and temperate wetlands [68]. In the Misiliscemi area, it is widely distributed and has integrated into both the landscape and local culture, being extensively used from a traditional perspective for weaving, construction, and other purposes [69].

The ecological profile of the flora assessed using Ellenberg Indicator Values (EIVs) perfectly reflects the characteristics of the plant landscape in the study area. The overall mean indicator values (Table 1), high for Light (L = 8.64) and Temperature (T = 8.28) and low for Moisture (U = 3.4), accurately describe the preference for high light exposure and a warm, arid Mediterranean climate. These values are characteristic of a flora typical of open, sunny environments, such as grasslands and scrublands widespread in the territory. Low Nutrient (N = 3.33) values are indicative of flora adapted to moderately nutrient-poor environments, common in semi-natural, minimally disturbed habitats. This is further supported by the comparison of EIVs between native and alien species (Table 1). Native species (N = 3.26) are more frequently found in nutrient-poor environments, whereas alien species (N = 3.81) thrive in richer soils. The difference between native and alien species (−0.55) is particularly pronounced, highlighting that alien species are associated with soils with greater nutrient availability. The same pattern is observed for Temperature, where the difference between native and alien species (−0.50) indicates that alien species thrive in warmer environments, while native species prefer slightly less warm climates. It is precisely the open, warmer environments composed of grassland mosaics, which in the lower-lying areas support ephemeral wetlands found between the districts of Chinisia and Marcanzotta (Figure 10), that serve as habitats for species of high naturalistic value.

## 5. Conclusions

The floristic indicators discussed have provided an understanding of the state of biodiversity, describing the landscape characteristics of this portion of the Sicilian territory. The detailed floristic inventory represents a fundamental scientific basis for biodiversity conservation and land management in the municipality of Misiliscemi, which, as a newly established administrative entity, requires accurate environmental data.

The analysis revealed that, within the rural-urban matrix, remnants of widespread naturalness persist, acting as refuges for native habitats and species, including rare and taxa of conservation interest. This hidden floristic diversity must not only be preserved but also actively enhanced. Furthermore, the floristic analysis identified the main environmental pressures, including arson-induced wildfires, invasive alien species, and the abandonment of traditional land management practices. To mitigate these threats, the implementation of municipal-level measures will be necessary, such as regulating the control and use of alien species and activating the Wildfires Registry (pursuant to Law No. 353/2000), which imposes post-fire restrictions (e.g., bans on grazing, hunting, and new construction) to counteract arson-related disturbances. Additional measures could be integrated into the management plans of the Natura 2000 Network, with targeted actions to reduce habitat pressures, or through regional incentives within the Rural Development Plan, aimed at restoring traditional agricultural systems.

Only through multi-level planning that integrates biodiversity knowledge into development strategies and actions can this young municipality achieve a sustainable balance between environmental conservation and socio-economic growth, ensuring the protection of its natural heritage and the well-being of local communities.

## Figures and Tables

**Table 1:** Comparison of Ellenberg Indicator Values (EIVs) for overall flora, native species, and alien species.

Variable	Overall Flora	Native	Alien	Difference Native/Alien
Light (L)	8.64	8.68	8.33	0.34
Temperature (T)	8.28	8.22	8.71	−0.5
Continentality (C)	4.47	4.43	4.78	−0.35
Moisture (U)	3.4	3.39	3.48	−0.09
Soil Reaction (R)	5.4	5.42	5.29	0.12
Nutrients (N)	3.33	3.26	3.81	−0.55

**Table 2:** Habitat according to the EUNIS Terrestrial Habitat Classification 2021. Wetlands, Constructed, Industrial and Other Artificial Habitats, and Complexes, following the 2012 classification.

EUNIS Habitat	Area [ha]
**Coastal habitats**	**5.68**
Mediterranean and Black Sea rocky sea cliff and shore (N32)	5.68
**Wetlands**	**222.38**
Tall-helophyte bed (Q51)	1.44
*Phragmites australis* beds (Q511)	215.15
Halophile *Scirpus*, *Bolboschoenus* and *Schoenoplectus* beds (Q517)	5.78
**Grasslands**	**387.13**
Semi-dry perennial calcareous grassland (meadow steppe) (R1A)	231.65
Mediterranean closely grazed dry grassland (R1D)	21.56
Mediterranean tall perennial dry grassland (R1E)	100.92
Inland salt steppes and salt marshes (R6)	17.76
Mediterranean inland salt steppe (R61)	6.70
Mediterranean *Limonium* salt steppes (R611)	8.54
**Heathland, scrub and tundra**	**38.61**
Olive arborescent matorral (S5121)	4.45
*Calicotome* brush (S51J5)	1.68
*Chamaerops humilis* brush (S51M)	5.08
*Spartium junceum* scrub (S53)	1.16
Western *Cistus* garrigues (S613)	4.58
*Nerium oleander*, *Vitex agnus-castus* and *Tamarix galleries* (S931)	0.85
Unvegetated or sparsely vegetated gravel bars in the Mediterranean region (U72)	20.82
**Forest and other wooded land**	**35.66**
Mediterranean riparian *Populus forests* (T142)	0.58
Italian *Quercus coccifera* forest (T2132)	0.54
Other broadleaved deciduous plantations (T1H4)	29.45
Coniferous plantation of non site-native trees (T3M)	5.09
**Vegetated man-made habitats**	**7404.20**
Mixed crops of market gardens and horticulture (V12)	66.16
Medium-scale intensive unmixed crops (1-25ha) (V112)	2740.10
Turf sports fields (V313)	15.97
Traditional vineyards (V541)	5.94
Intensive vineyards (V542)	2987.24
Fruit orchards (V614)	15.46
*Olea europaea* groves (V621)	1557.57
Citrus orchards (V622)	15.76
**Constructed, industrial and other artificial habitats**	**1146.94**
Residential buildings of city and town centres (J1.1)	169.96
Residential buildings of villages and urban peripheries (J1.2)	128.78
Urban and suburban factories (J1.42)	42.26
Agricultural constructions (J2.4)	8.89
Greenhouses (J2.43)	4.73
Active opencast mineral extraction sites, including quarries (J3.2)	2.88
Road networks (J4.2)	28.11
Rail networks (J4.3)	14.43
Airport runways and aprons (J4.4)	303.98
Saltworks (J5.1)	65.54
Saline and brackish industrial lagoons and canals (J5.11)	1.11
Non-agricultural organic waste (J6.3)	6.25
Intensively-farmed crops interspersed with strips of natural and/or semi-natural vegetation (X07)	370.03

**Table 3:** European Union habitats (Annex I, Directive 92/43/EC).

Legenda	Area [ha]*
1130: Estuaries	1.85
1150 *: Coastal lagoons	67.98
1210: Annual vegetation of drift lines	0.30
1240: Vegetated sea cliffs of the Mediterranean coasts with endemic *Limonium* spp.	4.28
1310: *Salicornia* and other annuals colonizing mud and sand	3.29
1410: Mediterranean salt meadows (*Juncetalia maritimi*)	11.97
1420: Mediterranean and thermo-Atlantic halophilous scrubs (Sarcocornetiea fruticosi)	18.75
1510 *: Mediterranean salt steppes (*Limonietalia*)	0.35
3280: Constantly flowing Mediterranean rivers with *Paspalo-Agrostidion* species and hanging curtains of *Salix* and *Populus alba*.	0.00
3290: Intermittently flowing Mediterranean rivers of the *Paspalo-Agrostidion*	20.82
5330: Thermo-Mediterranean and pre-desert scrub	9.94
6220 *: Pseudo-steppe with grasses and annuals of the *Thero-Brachypodietea*	116.31

* The surface areas in hectares were calculated based on the official habitat map of the Environmental Department of the Sicilian Region [49].

## Data Availability

The original contributions presented in the study are included in the Supplementary Materials; further inquiries can be directed to the corresponding author.

## Supplementary Materials

The following supporting information can be downloaded at: https://www.mdpi.com/article/10.3390/plants14040548/s1, Table S1: The vascular flora of Misiliscemi (Sicily).
